# Ullrich Congenital Muscular Dystrophy (UCMD): Clinical and Genetic Correlations

**Published:** 2013

**Authors:** Bita BOZORGMEHR, Ariana KARIMINEJAD, Shahriar NAFISSI, Bita JEBELLI, Urtizberea ANDONI, Corine GARTIOUx, Celine LEDEUIL, Valérie ALLAMAND, Pascale RICHARD, Mohammad-Hassan KARIMINEJAD

**Affiliations:** 1 Kariminejad-Najmabadi Genetic Center, Tehran-Iran; 2 Shariati Hospital, Tehran, Iran; 3 Pediatric Neurologist, Tehran, Iran; 4 Hospital Marin, Paris, France; 5 UPMC Univ Paris 06, IFR14, Paris, F-75013, France; 6 CNRS, UMR7215, Paris, F-75013, France; 7 Inserm, U974, Paris, F-75013, France; 8 Institut de Myologie, Paris, F-75013, France; 9 AP-HP, Groupe Hospitalier Pitié- Salpêtrière, UF Cardiogénétique et Myogénétique, Service de Biochimie Métabolique, Paris, F-75013, France

**Keywords:** Ullrich congenital muscular dystrophy, Collagen type VI, COL6A1, COL6A2

## Abstract

**Objective:**

Ullrich congenital muscular dystrophy (UCMD) corresponds to the severe end of the clinical spectrum of neuromuscular disorders caused by mutations in the genes encoding collagen VI (COL VI). We studied four unrelated families with six affected children that had typical UCMD with dominant and recessive inheritance.

**Materials & Methods:**

Four unrelated Iranian families with six affected children with typical UCMD were analyzed for COLVI secretion in skin fibroblast culture and the secretion of COLVI in skin fibroblast culture using quantitative RT–PCR (Q-RT-PCR), and mutation identification was performed by sequencing of complementary DNA.

**Results:**

COL VI secretion was altered in all studied fibroblast cultures. Two affected sibs carried a homozygous nonsense mutation in exon 12 of COL6A2, while another patient had a large heterozygous deletion in exon 5-8 of COL6A2. The two other affected sibs had homozygote mutation in exon 24 of COL6A2, and the last one was homozygote in COL6A1.

**Conclusion:**

In this study, we found out variability in clinical findings and genetic inheritance among UCMD patients, so that the patient with complete absence of COLVI was severely affected and had a large heterozygous deletion in COL6A2. In contrast, the patients with homozygous deletion had mild to moderate decrease in the secretion of COL VI and were mildly to moderately affected.

## Introduction

Congenital muscular dystrophy (CMD) constitutes a heterogeneous group of disorders characterized by muscle weakness and hypotonia at birth (1). These disorders vary in severity, symptoms, outcomes, pathological findings, and genes (1).

In 1930, Ullrich described two unrelated patients with CMD, and the disorder was described as scleroatonic muscular dystrophy with peculiar clinical findings (2,3). The clinical manifestations of UCMD are muscle weakness of early onset, proximal joint contractures, distal hyperextensibility, and normal intelligence (2,3).

Decreased fetal movement is often noted in the prenatal period. Muscle weakness is usually severe and, patients typically never walk independently, or do so only for a short time (1,4,5) Over time, there is development of spinal rigidity and kyphoscoliosis and variable contractures in proximal joint, distal joint hyperlaxity, and tight Achilles tendons (1,4,5).

In the first or second decade of life, respiratory failure has been reported as a common cause of death unless treated by nocturnal respiratory support. Failure to thrive is common. Cardiac involvement is generally absent (1,4-6). Other distinctive features observed in UCMD patients are as follows: congenital hip dislocation, torticollis, multiple joint contractures of knees and elbows, kyphotic contracture of the spine, and skin changes, such as hypertrophic scars and hyperkeratosis follicularis (1,4-6). Serum creatine kinase (CK) levels are usually normal or mildly increased (4). Electromyography (EMG) shows action potentials of low amplitude and short duration (7). Variable pathology ranging from non-specific mild myopathic changes to more dystrophic-like changes, can be observed in muscle biopsies of UCMD patients. The spectrum includes variation in fibre size, type 1 fibre predominance, increase in endomysial connective tissue, increase in numbers of internal nuclei, and focal areas of necrosis, along with more indirect evidence of muscle fibre regeneration, including the presence of fibres containing fetal myosin (1,4,8,10).

This disorder was originally identified as a recessive condition with homozygous and compound heterozygous mutations in one of the three genes (COL6A1, COL6A2, and COL6A3) encoding three chains, which are assembled and secreted to form collagen VI (ColVI) microfibrils. Nevertheless, over the last few years, a number of patients presenting with clinical manifestations of severe UCMD were shown to carry heterozygous mutations in these genes, which were not transmitted but acquired as dominant de novo (9). UMCD, [MIM] # 254090 and Bethlem myopathy (BM), [MIM] # 158810, are two extremes of a contiguous clinical spectrum, which intermediate observed phenotype (14). Classical UCMD is the severe end and classical BM corresponds to a milder form with late onset and static course (5).

## Materials & Methods

We studied six affected children (5 males, 1 female) from four unrelated Iranian families. All of them were diagnosed with a classical UCMD Phenotype. The parents were consanguineous, healthy, and normal according to the clinical examination. According to ethical rules, all parents signed an informed written consent for photography, genetic analysis, blood sampling, and skin biopsy.

**Fig 1:**
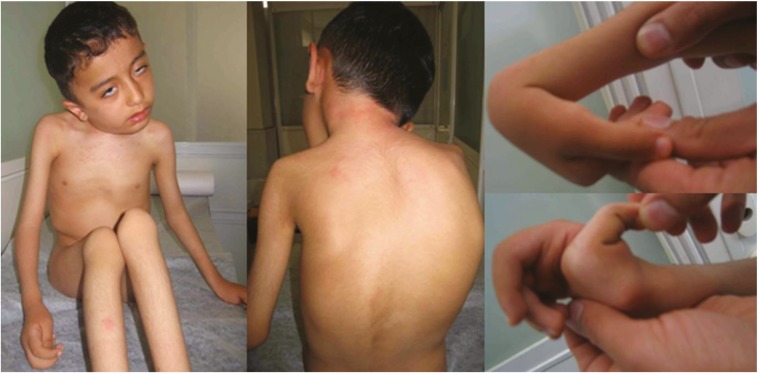
Family A: a, torticollis; b, scoliosis; c, hyperextensibility of wrist; d, hyperextensibiliy of fingers

The affected child from family A was a 6-year-old boy. His mother reported decreased fetal movement during pregnancy. He was born at 39 weeks of gestation by cesarean section, due to breech position. His birth weight, length, and head circumference were all within normal limits (2800 gram, 48, and 34 cm, respectively). At birth, the patient presented with severe hypotonia, contractures in knees, and flexion deformity of elbows and rigidity of spine. At the age of 10 months, he showed limited movements of shoulder and hip and hyperextensibility of fingers, wrists, toes, and ankles. Torticollis, stiff neck, and severe kyphoscoliosis were also noted as the disease progressed. He could sit only with support at the age of 2 only for six months and never achieved the ability to stand and walk. His first visit at our centre was at the age of 6 years. He showed severe clinical manifestations of UCMD with normal intelligence. All biochemical, endocrinological, and metabolic tests were normal except for a slight increase of CK levels [255 IU/L (Normal range: 50-200 IU/L)]. EMG showed a chronic myopathic process. The child progressively developed respiratory failure, underwent tracheostomy, and was eventually put on nocturnal positive pressure mechanical ventilation at the age of 8. However, he passed away six months later due to pneumonia (Figure 1).

Family B had two affected and one healthy offsprings. The first child was a healthy aged 12-year-old girl. The second child was a 10-year-old affected girl, and the third was a 7-year-old affected boy. Both affected children were born at 40 weeks of gestation following normal vaginal delivery. Their birth weight, head circumference and height were within normal limits.

Hypotonia and knee stiffness were noted at birth. They were examined in our centre when the girl was 10 and the boy was 7 years old. They could sit with support and their cognition was normal. Both of them had torticollis, stiff neck, limited movement of elbows and knees, kyphoscoliosis, and hyperextensibility of wrists, fingers, ankles, and toes. They never achieved ability to stand and walk independently.

All biochemical, endocrinological, and metabolic tests were normal. EMG showed a chronic myopathic process in both. They are still alive after three years with a slow progression in skeletal deformity.

Family C had two affected sons. The first son was 7 years old and the second son was 2 year old. Both of them were born at 40 weeks of gestation following normal delivery. Their birth weight, head circumference, and height were within normal limits. They were hypotonic at birth without any contractures. They were examined in our center when the first son was 7 and second was 2 year old. The older son could walk with support for 2 years from 4 to 6 years of age, but he lost the ability of walking after 6. The younger son has not been able to walk yet. Their cognition was normal. The older son had contracture of elbows and knees as well as torticollis, scoliosis, and hyperlaxity of fingers, toes, wrist, and ankle,. The younger son had hyperextensibility of wrist, fingers, toes and knees, without any contractures in joints. All biochemical, endocrinological and metabolic tests were normal. EMG of the older son showed a chronic myopathic process. They are still alive after one year.

Family D had a 11-year-old affected son. His mother reported decreased fetal movements. Birth weight, length, and head circumference were all within normal limits (2900 gram, 50 cm, and 34 cm, respectively). At birth, the patient presented with severe hypotonia without any contractures. He was examined in our center at the age of 11. He could sit with support at the age of 12 months, but he never achieved ability to walk. His cognition was normal. He had contracture of shoulders, hips, knees, and ankles, which were first noted at 4-5 years. He had hyperextensibility in fingers but not in toes. He also had an ulnar deviation in his wrist, and hyperkeratosis follicularis. All biochemical, endocrinological, and metabolic tests were normal EMG showed chronic myopathic process.

**Collagen VI immunolabeling on cultured skin fibroblasts**

Muscle cryosections were not available, so, we did skin biopsy for six patients. ColVI immunolabeling were carried out on the skin fibroblasts of one control and six patients. Col VI secretion was investigated on confluent fibroblasts cultured in the presence of 0.25 mM ascorbic acid for 5 days, then, stained with the MAB 1944 antibody (Chemicon International), diluted 1:100, and incubated overnight at +4°C.

Slides were observed using an Axioplan 2 microscope (Zeiss) equipped with a HBO 100 mercury lamp (Zeiss) and representative images were obtained by the Metaview software.

**Fig 2:**
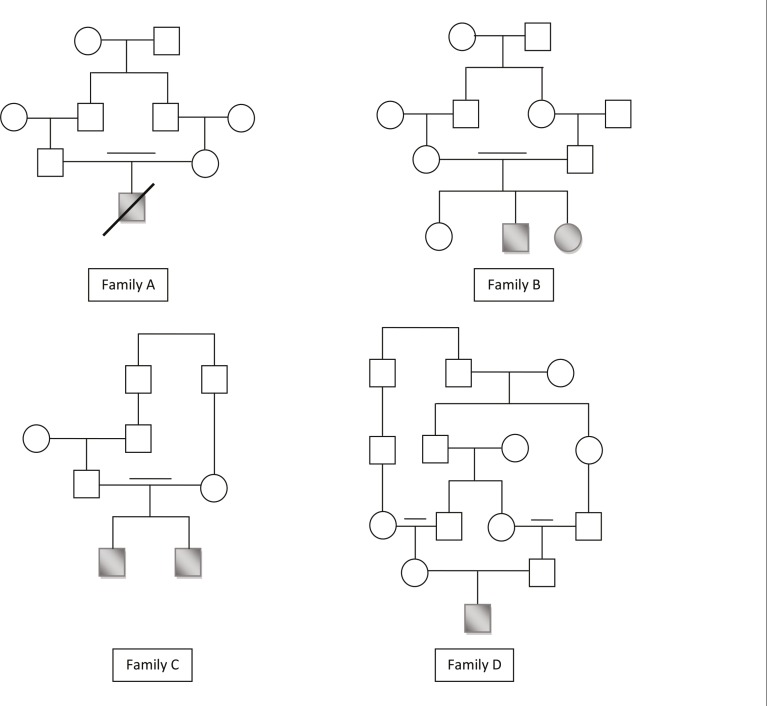
Pedigrees of UCMD families

**RNA Extraction and Quantitative RT-PCR**

Total RNA was extracted from confluent fibroblast cultures using Trizol® (Invitrogen) according to the manufacturer’s recommendations. cDNA prepared from 1 µg of total RNA using oligo (dT) 18 and superscript II reverse transcriptase as recommended by the manufacture. Chain specific transcripts were quantified from fibroblast-derived cDNA using quantitative RT-PCR on a LightCycler via primers hybridizing to the last C-terminal of each domain.

**Genetic analysis**

Genomic DNA from patients and their parents was obtained from blood leukocytes by standard methods.

The coding regions of the genes were amplified from fibroblast-derived cDNA in several overlapping fragment (primers available on request), and sequenced on the ABI3730 capillary electrophoresis system. Sequences were compared with reference sequences, NM-001848.2 (COL6A1), NM-001849.3 (COL6A2), and NM-004369.3 (COL6A3). Each potentially pathogenic change was confirmed on genomic DNA from patient and their parents. All tests were done in GIS Institute in Paris. homozygous deletion of exon 24 [c.1771-1819 del] causing a shift in the reading frame at codon Glu591 and creation of a premature stop codon at position [p.Glu591ThrfsX149].

In family D, a pathogenic mutation was identified that consisted of 259 base pairs of exon 19 in the cDNA and resulted in a frame shift in the reading frame, and in homozygote mutation of COL6A1. All Mutations was confirmed on genomic DNA from the patients

**Table 1:** Primers Used in Quantitative RT-PCR

Gene	Forward (5’ → 3’)	Reverse (5’ → 3’)
COL6A1	TCAGAGAGTACTCGCAGGGG	AGAAAGGGGGCCTTTGATAA
COL6A2	CTGCACCTCTCCAGCTCCT	AAGCCTTTATTGGGTCAGGG
COL6A3	CGAAAGACGAAGGAACTTGC	CACCCGGAGCTTCTACAGAG

## Results

In four studied families, transcript levels of each gene were quantified using specific primers and revealed a distinct reduction of the COL6A2 mRNA species in the three families, while level of the COL6A1 was reduced in family D. COL6A3 mRNA were within the normal range in all families. These data clearly suggest that COL6A2 and COL6A1 gene carry the pathological mutations in these families.

Sequencing of mRNA from the affected boy of family A revealed a large heterozygous deletion encompassing exons 5 to 8 of the COL6A2 transcript. No additional mutations were detected in COL6A1, COL6A2 and COL6A3 genes. The deletion was not present in the parents, thus, the COL6A2 deletion occurred de novo. Concerning family B, sequencing of the coding regions of the COL6A2 gene revealed a homozygous nonsense mutation in exon 12 (c.1096 C>T) leading to a premature termination codon (p.R366X), in both siblings and at the heterozygous state in both parents. The occurance of the premature stop codon most likely triggered the nonsense mediated mRNA decay (NMD) pathway leading to the degradation of the corresponding transcripts and the low levels observed by quantitative RT-PCR.

In family C, analysis of cDNA sequence showed a and their parents. COLVI expression and secretion pattern in skin fibroblast cultures was normal in control fibroblasts and abnormal secretion was detected in all the patients’ fibroblast cultures.

**Fig 3:**
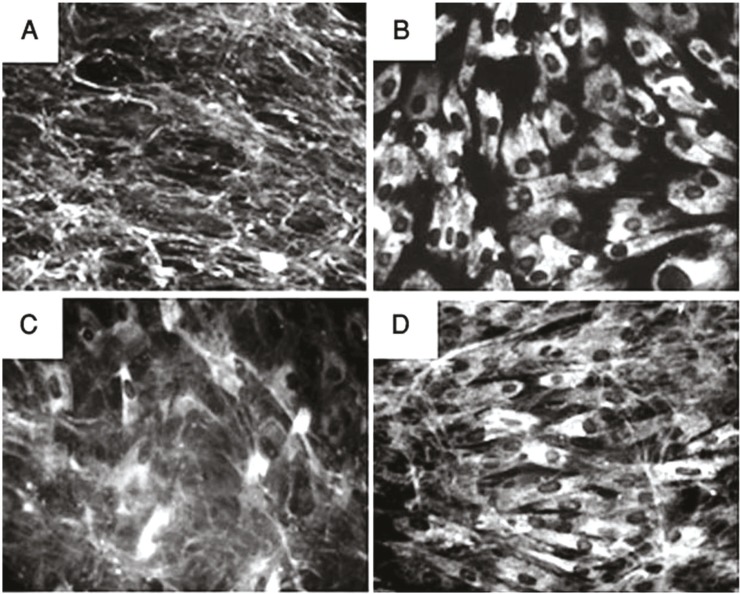
Collagen VI (COL VI) immunolabeling of control and patients derived cultured fibroblasts (A) Control fibroblasts formed a dense COLVI net. (B, C, D) COLVI immunolabeling in fibroblasts from patients as examples of complete secretion deficiency, strongly reduced secretion, reduced secretion, respectively

COLVI secretion was completely absent in first patient, strongly reduced in family B and slightly reduced in family C and D (Figure 3 and supporting information in Table 2).

## Discussion

UCMD is a disorder characterized by muscle weakness, proximal joint contractures, and hyperlaxity of distal joints. It has long been considered to follow an autosomal recessive mode of inheritance. The pattern of inheritance was challenged when patients with severe clinical findings compatible with UCMD were shown to have heterozygous deletions in COL6A1, COL6A2, or COL6A3. The first case of autosomal dominant inheritance was reported by Pan et al. (2003). The patient had a de novo heterozygous deletion of COL6A2 gene that resulted in a severe phenotype of UCMD, however, that particular deletion was not present in their parents (9). Baker et al. (2005) reported three patients with heterozygous deletions in the COL6A1, COL6A2, COL6A3 genes. The study showed that these mutations act in a dominant mode and result in severe collagen VI deficiency (10).

**Table 2:** Clinical, Genetic and Immonolabeling Data

**Patient**	**Sex** **(age)**	**Disease** **severity**	**Motor** **capacity** **(Year)**	**Congenital** **Contracture**	**Skin** **Changes**	**Spinal** **deformity**	**Col VI** **secretion in** **fibroblasts** **culture**	**Gene**	**Inheritance**
A1	M(6y)	Very severe	Sits at the age of 2 for six month	Yes	Yes	Yes	Completely absent	Col 6A2del exon 5 to 8	AD de novo Heterozygote
B1	F(10)	Moderate to severe	Sits with support from age of 2	Yes	No	Yes	Strongly reduced secretion	Col 6A2 del exon 12	AR Homozygote
B2	M(7y)	Moderate to severe	Sits with support from age of 2	Yes	No	Yes	Strongly reduced secretion	Col 6 A2 del exon 12	AR Homozygote
C1	M(7y)	Mild to Moderate	Walks with support from age 4 to 6	No	No	Yes	reduced secretion	Col 6 A2 del exon 24	AR Homozygote
C2	M(2y)	Mild to Moderate	Sits with support from age of 1	No	No	Yes	reduced secretion	Col 6 A2 del exon 24	AR Homozygote
D1	M(11y)	Moderate	Sits with support from age of 1	No	Yes	Yes	reduced secretion	Col 6 A1	AR Homozygote

Pace et al. (2008) reported 8 patients with heterozygous collagen VI mutations. All eight patients had heterozygous glycine mutations in the triple helix. These anomalies have already been associated with Bethlem and UCMD phenotypes (11).

BM was first described in 1976 by Bethlem and Wijngaarden and was considered as an autosomal dominant condition. BM is generally a mild and late proximal myopathy with finger flexion contractures (12). Gualandi et al. (2009) and Foley et al (2009) both reported patients with BM with a recessive mode of inheritance (13,14).

It is known that BM and UCMD represent opposite ends of a COLVI related disorders spectrum, in which patients with intermediate phenotypes could be considered to have either severe UCMD or mild BM (13).

BM is a mild disorder with major impact on adult life. Patients may have neonatal hypotonia or delayed motor milestones that can become symptomatic within the first or second decade of life. However, some adult patients remain unaware of weakness until late age (12).

BM is transmitted both as autosomal dominant and recessive disorder. It is associated with mutations in COL6A1, COL6A2, and COL6A3 (13).

UCMD has been considered a recessive condition with homozygous or compound heterozygous mutations in COL6A1, COL6A2, and COL6A3. However, studies have shown that heterozygous de novo deletions in COL6A1, COL6A2, and COL6A3 cause both severe UCMD and mild BM (13-15). Paceetal.indicatedthatCOL6mutationsimpairtheCOLVI synthesis pathway in different ways, and disease severity correlates with the assembly abnormality. In mildly affected patients, normal amounts of collagen VI were deposited in the fibroblasts, while in patients with severe disability, collagen VI was reduced in fibroblasts (11). Here we present 6 patients with early onset COLVI myopathy with a variable severity of presentation and progression, and harbouring mutations in one of the CoLVI encoding genes. Our first patient with large heterozygous deletion in exon 5 to 8 on COLA2 and absent secretion of COLVI in fibroblasts had severe contractures, respiratory failure with rapid progression to death. The siblings in the second family with homozygous deletion in exon 12 of COLA2 and strongly reduced COLVI secretion in fibroblasts had moderate phenotype of UCMD.

In Contrast, the siblings of the third family with homozygous deletion of exon 24 of COL6A2 and mild reduced COLVI secretion in fibroblasts, had milder phenotype of UCMD with slow progression. The last patient in the fourth family was homozygote in COL6A1 and showed very slow progression of disease.

**In conclusion**, taking together all the results presented above, we found a correlation between the clinical severity and COLVI secretion in cultured fibroblasts, and genotype.

The patient with complete absence of COLVI secretion was severely affected, and had a large heterozygous deletion in COL6 A2.

In contrast, the patients with homozygous deletions were mildly to moderately affected. More investigations should be performed in the future to show the mechanism of differences between autosomal recessive UCMD, autosomal dominant UCMD with severe phenotype, and autosomal dominant BM with mild phenotype.
